# Cardiac Phase Space Analysis: Assessing Coronary Artery Disease Utilizing Artificial Intelligence

**DOI:** 10.1155/2021/6637039

**Published:** 2021-04-09

**Authors:** Mark G. Rabbat, Shyam Ramchandani, William E. Sanders

**Affiliations:** ^1^Loyola University Medical Center, USA; ^2^CorVista Health, Toronto, Ontario, Canada; ^3^University of North Carolina at Chapel Hill, Chapel Hill, North Carolina, USA; ^4^CorVista Health, Cary, North Carolina, USA

## Abstract

The bridge of artificial intelligence to cardiovascular medicine has opened up new avenues for novel diagnostics that may significantly enhance the cardiology care pathway. Cardiac phase space analysis is a noninvasive diagnostic platform that combines advanced disciplines of mathematics and physics with machine learning. Thoracic orthogonal voltage gradient (OVG) signals from an individual are evaluated by cardiac phase space analysis to quantify physiological and mathematical features associated with coronary stenosis. The analysis is performed at the point of care without the need for a change in physiologic status or radiation. This review will highlight some of the scientific principles behind the technology, provide a description of the system and device, and discuss the study procedure, clinical data, and potential future applications.

## 1. Background

Cardiovascular disease is the leading cause of death worldwide. Thus, accurate diagnosis in patients with suspected coronary artery disease (CAD) is critical in clinical medicine. For the majority of patients, standard of care assessment for CAD begins with a functional stress test. In the United States alone, millions of stress tests are performed on an annual basis to evaluate patients with suspected CAD. However, this pathway has been reported to have low diagnostic yield at the time of invasive coronary angiography (ICA) [1]. Obstructive CAD was noted in less than half of patients undergoing exercise treadmill testing, stress echocardiography, single-photon emission computed tomography (SPECT) imaging, and stress cardiac magnetic resonance imaging at the time of their ICA in a contemporary analysis from the National Cardiovascular Data Registry (NCDR) of more than 385,000 patients from >1,100 United States hospitals [2]. Noninvasive testing has demonstrated similar prediction of obstructive CAD compared to clinical factors [2]. Moreover, a recent study of over 15,000 patients found that among patients referred for ICA, those with a positive stress test were less likely to have obstructive CAD and receive revascularization compared to those either with a negative stress test or no testing at all [3].

The bridge of artificial intelligence to cardiovascular medicine has opened up new avenues for novel cardiovascular diagnostics that may significantly enhance the care of patients [4–6]. Unlike traditional imaging modalities to assess for CAD, cardiac phase space analysis (cPSA) is a dynamic assessment that captures data related to electrical signals over consecutive cardiac cycles which is unique to a given individual [7]. The resultant thoracic phase signals are analyzed by cPSA to quantify physiological and mathematical features associated with coronary stenosis without the need for a change in physiologic status such as stress-induced vasodilation.

cPSA is an easy-to-use, portable device utilized at the point-of-care without radiation, contrast, or patient preparation. This review will highlight some of the scientific principles behind the technology, provide a description of the system and device, and discuss the study procedure, clinical data, and potential future applications.

## 2. Cardiac Phase Space Analysis

### 2.1. Signal Acquisition

**Figure 1:**
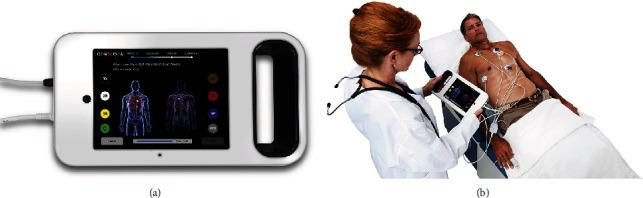
Data acquisition setup: (a) signal acquisition device and (b) patient and electrodes/lead placement and PPG configurations.

Two sources of time series data are simultaneously acquired from each subject: (i) orthogonal voltage gradient (OVG) signals and (ii) photoplethysmography (PPG) signals. These signals are collected with a sampling rate of 8 kHz using a specialized instrument (both hardware and firmware), shown in Figure 1. Signals are acquired for 3.5 minutes, resulting in a short overall procedure time, conducive to an outpatient-based single visit clinical assessment. Signal quality scores quantified nonbiological interference that could affect the performance of subsequent analyses. The OVG signal is assessed for powerline interference (60 Hz based on the main frequency in North America) and excessive frequency content greater than 170 Hz (high-frequency noise). Additionally, the quality of the PPG signal is assessed through quantifying the segments of the signal affected by jumps and dropouts (abrupt jump noise) and epochs that do not have dynamic variations reflecting the change in the blood flow volume changes (railing noise). Signals exceeding the threshold for any of the described scores are excluded, and signals passing the quality assessment are preconditioned by removing baseline wander and filtering the high-frequency noise and powerline interference.

### 2.2. Photoplethysmography (PPG)

PPG is used to optically measure the variations of the volume of blood perfusing the tissue. In this measurement modality, a specific wavelength of light is emitted from an LED illuminating the tissue (e.g., skin, subcutaneous tissue, and fat); the intensity of this light after passing through the tissue (in this case, fingertip) is then registered by photodetectors. The amount of light absorbed by the interrogated tissue depends on the volume of the blood. This variation is observed in the PPG signals and can provide valuable information with regard to, among other things, to cardiac activity.

The PPG signals can be used for various purposes such as monitoring the blood oxygen saturation level when two light sources are used as well as for measuring and analyzing heart rate variability. The PPG signals are recorded using a sensor with red and near-infrared light sources. These PPG waveforms are then employed for analysis and feature extraction.

### 2.3. Orthogonal Voltage Gradient (OVG)

The three-dimensional OVG measures the electrical activity, the product of the action potential generation, of the heart. There are various configurations of the leads that can be used to obtain such signals. With the signal acquisition device configuration shown in Figure 1, seven leads are used which result in three orthogonal channels, denoted *X*, *Y*, and *Z*. These signals are measured in the patient's coronal, sagittal, and transverse planes, respectively.

### 2.4. Machine Learning

Measurements of the signals are made using Phase Space mathematics and other mathematical approaches such as dynamical system analysis to create a set of measurements or features. These features are then paired with the corresponding “ground truth labels” (actual catheterization results) to form the input to the machine learning (ML) models. Many types of ML models can be applied to these data (Random Forests, Neural Networks, Genetic Algorithms, and Support Vector Machines), but the choice(s) of ML method can drive specific settings of the data set and parameters to be evaluated. A standard example of a ML campaign: the data are split into training-validation and test sets (usually 80% training 20% validation but this can be adjusted from campaign to campaign). The training-validation set is used to train and fine-tune several machine learning models using 5-fold cross-validation. To find an optimal set of hyperparameters for each model, a grid search is performed over a range of hyperparameters. Then, using the average AUC of 100 runs as the performance metric, the set of hyperparameters that results in the highest validation AUC is selected for each model. The models are ranked by performance on the validation dataset. In the final step, the selected models are trained on the entire train-validation set, and their AUC performance on the held-out naïve test set is assessed.

### 2.5. System and Device Description (Figure 1)

The cPSA System is a medical device system that uses novel features and machine-learned algorithms to analyze phase signals and assess the presence of significant epicardial CAD. The first element is the Phase Signal Acquisition (PSAQ) System. The PSAQ includes the phase signal recorder (PSR) and the phase signal data repository (PSDR). The PSR is a hand-held instrument that acquires and transmits resting phase signals along with additional patient-specific information such as gender and age. The cloud-based PSDR accepts, stores, and allows retrieval of the signals as well as patient-specific information. The second element is a CAD analytical engine (CAD AE). Utilizing machine-learned algorithms, the CAD AE processes and evaluates the phase signals from approximately 10 million data points to assess the presence and significance of CAD. The final element is the health care provider (HCP) Web Portal that the clinician utilizes to interpret images, review results, and generate a report. The results are subsequently displayed as a phase space analysis model, and the report can be saved as a record for inclusion in the patient's electronic medical record.

### 2.6. Study Procedure

Signals are acquired utilizing the hand-held PSR device via seven sensors positioned on the chest and back and a PPG sensor clipped to a finger. Phase signal data are collected for approximately 3 minutes, and the data is then transmitted wirelessly to the cloud based PSDR. An analytic engine, consisting of software based on the machine-learned algorithms, analyzes the acquired data and generates predictions of physiological status. The results are made available through a secure web portal.

### 2.7. Clinical Data

**Figure 2:**
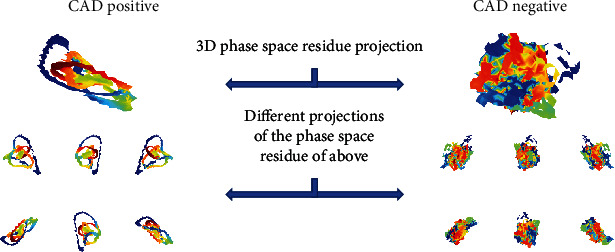
Phase Space (PS) Residues from a CAD positive subject and CAD negative subject. The PS Residues are 3D computation objects generated from the difference of the actual signal from the modelled signal in three dimensions. These objects can be evaluated geometrically to produce features (such as surface area or volume). The coloring can represent another measurable dimension. Here, the images are colored by where in the depolarization/repolarization cycle the point difference comes from. The top image is a single projection of the 3D PS Residue image. The 6 smaller projections are different views of the larger object.

The primary objective of the Coronary Artery Disease Learning and Algorithm Development (CADLAD) trial was designed to collect resting phase signals from eligible subjects using the PSR prior to ICA to machine learn and test an algorithm for detecting the presence of significant CAD in symptomatic patients [7]. In addition, machine-learned algorithms were developed and tested to identify the location of significant CAD. Demographics and patient characteristics for the studied population are shown in Table 1. With the aim of a generalized machine-learned algorithm in mind, a broad cross section of clinical practices at twelve enrolling centers throughout the United States, representing a diverse array of facilities providing care to patients with heart disease, were utilizedas investigational sites. First, OVG signals were paired with clinical outcomes data to develop machine-learned algorithms for the assessment of significant CAD. Subsequently, a blinded paired comparison of the machine-learned algorithm was performed against the “gold standard” (ICA) for assessment of CAD. Significant CAD was defined as a diameter reduction ≥ 70% or at least one lesion with reduced fractional flow reserve (FFR) of ≤0.80 at the time of ICA. Initial results from the CADLAD trial included 606 participants. The machine-learned algorithm cohort consisted of phase signals from 512 patients with 94 patients serving as the verification cohort. Blindly testing the cPSA System in the naïve verification cohort demonstrated a sensitivity of 92% (95% CI: 74%-100%) and specificity of 62% (95% CI: 51%-74%) for the assessment of significant CAD, which is comparable to commonly performed standard of care functional testing (Table 2) [7–9]. The negative predictive value (NPV) was 96% (95% CI: 85%-100%), and the PPV was 46% (95% CI: 33%-62%) [7]. In order not to miss significant CAD in clinical practice, the system was optimized (threshold chosen using the AUC-ROC curve) to maximize safety and therefore sensitivity. The specificity of 62% remains comparable to other functional tests [7].  Figure 2 presents cases of patients with and without CAD.

Conventional diagnostic pathways for detecting CAD are less accurate in women than men. Preliminary data from the CADLAD trial revealed that the diagnostic performance of cPSA for women compared to men was equivalent if not superior demonstrating an overall area under the receiver-operator characteristic curve (AUC) (0.82 (0.60-0.96) vs. 0.76 (0.62-0.86)), sensitivity (100% (100%-100%) vs. 83% (56%-95%)), specificity (73% (42%-92%) vs. 64% (49%-76%)), and NPV (100% (100%-100%) vs. 91% (76%-97%)), respectively (*p* = ns for all).

When stratified by age, initial data from the CADLAD trial demonstrates comparable diagnostic performance of cPSA for those <65 years of age and ≥65 years of age with an overall AUC (0.79 (0.66-0.88) vs. 0.72 (0.50-0.88)), sensitivity (100% (100%-100%) vs. 86% (56%-100%)), specificity (63% (49%-75%) vs. 67% (40%-88%)), and NPV (100% (100%-100%) vs. 83% (50%-100%)), respectively (*p* = ns for all).

In addition, those with obesity (body mass index ≥ 30 kg/m^2^) had similar diagnostic performance with cPSA compared to subjects without obesity (body mass index < 30 kg/m^2^) demonstrating an overall AUC (0.78 (0.64-0.88) vs. 0.80 (0.62-0.92)), sensitivity (83% (46%-100%) vs. 92% (50%-100%)), specificity (67% (51%-79%) vs. 67% (44%-84%)), and NPV (94% (79%-100%) vs. 94% (68%-100%)), respectively (*p* = ns for all).

SPECT is the most ubiquitous functional stress test performed in the United States. In the CADLAD trial, SPECT was performed in a subgroup of 607 subjects prior to their ICA. Positive SPECT results were compared to the machine-learned cPSA algorithm using ICA as the reference standard. Overall sensitivity (86% (81%-91%) vs. 92% (86%-96%)), specificity (23% (19%-27%) vs. 33% (27%-39%)), PPV (36% (32%-41%) vs. 42% (36%-48%)), and NPV (77% (68%-84%) vs. 89% (81%-95%)) were comparable between SPECT and cPSA, respectively (*p* = ns for all).

### 2.8. Future Directions

The bridge of AI utilizing cPSA and cardiovascular medicine has a very bright future. The same principles and methods developed for assessment of CAD can be utilized for other cardiovascular conditions. Ongoing clinical research with cPSA in pulmonary hypertension and left ventricular end diastolic pressure are underway. As a society, we need to assure these algorithms and others developed are used wisely. Thus, larger and more heterogeneous data sets are required in order to limit bias and increase the generalizability in patient populations such as women and minority groups [10–12].

## 3. Conclusion

Features extracted from thoracic phase signals can be employed in machine learning to develop final mathematical predictors that assess the presence of significant CAD. Performance of the *c*PSA appears comparable to the most commonly employed functional stress tests without the need for ionizing radiation, contrast media, or stress (exercise or pharmacological) and requires minimal patient time.

## Figures and Tables

**Table 1:** Demographics of population.

Characteristics	Development (*n* = 512)	Verification (*n* = 94)	*p* value
Mean age, years (range)	61.5 ± 10.7	59.0 ± 9.8	0.04
Male (%)	60.2%	69.1%	0.11
Female (%)	39.8%	30.9%	0.11
Mean BMI (range)	31.3 ± 7.0	32.5 ± 7.6	0.14
Diabetes mellitus (%)	31.4%	35.1%	0.47
Hypertension (%)	72.9%	75.5%	0.70
Hypercholesterolemia/hyperlipidemia (%)	71.3%	70.2%	0.90
Angiographic results = CAD negative (%)	69.1%	73.4%	0.46
Angiographic results = CAD positive (%)	30.9%	26.6%	0.46

Reproduced with permission (Stuckey TD, et al. PLOS ONE. 2018).

**Table 2:** Detecting flow-limiting CAD. Machine-learned predictor (cPSTA) compared to exercise SPECT [8] and exercise ECG [8, 9].

Test	Sensitivity range	Specificity range
Rest cPSTA (*N* = 94)^∗^	92% (95% CI = 74% to 100%)	62% (95% CI = 51% to 74%)
Exercise SPECT	82-88%	70-88%
Exercise ECG	54-75%	64-75%

Reproduced with permission (Stuckey TD, et al. PLOS ONE. 2018).
